# SAPV-Patienten in der COVID-19-Krise: Eine interviewbasierte Studie über Erfahrungen von Palliativpatienten

**DOI:** 10.1007/s00761-022-01189-6

**Published:** 2022-05-25

**Authors:** Ulrich Kaiser, Ursula Vehling-Kaiser, Martin Kalteis, Ana Hoffmann, Jörg Schmidt, Florian Kaiser

**Affiliations:** 1 Klinik und Poliklinik für Innere Medizin III, Universitätsklinikum Regensburg, Franz-Josef-Strauß-Allee 11, 93053 Regensburg, Deutschland; 2 Onkologisches und Palliativmedizinisches Netzwerk Landshut, Landshut, Deutschland; 3 SAPV Adiuvantes Landshut, Landshut, Deutschland; 4 VK&K Studien GbR Landshut, Landshut, Deutschland; 5 Institut für Marktforschung im Gesundheitswesen München, München, Deutschland; 6 Klinik für Hämatologie und medizinische Onkologie, Universitätsmedizin Göttingen, Göttingen, Deutschland

**Keywords:** COVID-19-Pandemie, Palliativpatient, Ambulante Palliativversorgung, Schutzmaßnahmen, Telemedizin, COVID-19 pandemic, Palliative patient, Outpatient palliative care, Protective measures, Telemedicine

## Abstract

**Hintergrund:**

COVID-19 betrifft im ambulanten Bereich vor allem auch Palliativpatienten, die im Rahmen der spezialisierten ambulanten Palliativversorgung (SAPV) versorgt werden. Zur Vermeidung von Infektionen wurde die Implementierung von neuen Sicherheitsvorkehrungen und telemedizinischen Kommunikationsmöglichkeiten in die an der Studie beteiligten SAPV erforderlich.

**Ziel der Arbeit:**

Die Studie untersucht die Auswirkungen der COVID-19-Pandemie auf die persönlichen und sozialen Probleme von Palliativpatienten und ihre Erfahrungen mit der betreuenden SAPV.

**Material und Methoden:**

20 SAPV-Patienten wurden in halbstrukturierten Telefoninterviews zu ihren Problemen im Zusammenhang mit der Pandemie und Erfahrungen mit der SAPV-Betreuung befragt.

**Ergebnisse:**

Angst vor Einsamkeit und Infektion belasten Palliativpatienten sehr. Die meisten Patienten wollten Krankenhausaufenthalte wegen erhöhter Infektionsgefahr vermeiden. Schutzmaßnahmen der SAPV gaben ihnen ein Gefühl der Sicherheit und wurden trotz Einschränkung des persönlichen Kontakts akzeptiert. Moderne Kommunikationsformen waren nützlich, konnten aber den persönlichen Kontakt nicht ersetzen.

**Diskussion:**

Die Pandemie führte zu Veränderungen in der SAPV und hatte Auswirkungen auf das soziale Umfeld von Palliativpatienten. Schutzmaßnahmen sind für das Sicherheitsgefühl der betreuten Palliativpatienten wichtig. Die Versorgungsqualität der an COVID-19 angepassten SAPV-Struktur wird von den Patienten meist nicht als verschlechtert wahrgenommen. Ängste vor sozialer Isolation nehmen bei den Palliativpatienten einen hohen Stellenwert ein und können durch die SAPV abgebaut werden. Der persönliche Kontakt zur SAPV kann durch moderne Kommunikationsmöglichkeiten nicht ersetzt werden, wobei die Patienten Telemedizin im Sinne einer „Notlösung“ durchaus akzeptieren.

## Abstract (en)

**Background:**

In the outpatient setting, COVID-19 primarily affects palliative care patients who receive care as part of specialized outpatient palliative care (SAPV). To prevent infections, the implementation of new safety precautions and telemedical communication options in the SAPV participating in the study became necessary.

**Objective:**

This study examines the impact of the COVID-19 pandemic on palliative care patients’ personal and social problems and their experiences with caregiving SAPV.

**Material and methods:**

A total of 20 SAPV patients were interviewed about their problems in relation to the pandemic and about their experiences with care provided by SAPV in semi-structured phone interviews.

**Results:**

Fear of loneliness and infection weigh heavily on palliative care patients. Most patients wanted to avoid hospitalization due to increased risk of infection. Protective measures of SAPV gave them a sense of security and were accepted despite restrictions on personal contact. Modern forms of communication were useful but could not replace personal contact.

**Conclusion:**

The pandemic led to changes in SAPV and had an impact on the social environment of palliative care patients. Protective measures are important for the sense of security of palliative care patients under care. The quality of care provided by the SAPV structure adapted to COVID-19 is usually not perceived by patients as having deteriorated. Fears of social isolation are highly valued by palliative care patients and can be reduced by SAPV. Personal contact with SAPV cannot be replaced by modern communication options, although patients do accept telemedicine as an “emergency solution”.

Ein Teil der Palliativpatienten in Deutschland wird über die spezialisierte ambulante Palliativversorgung (SAPV) betreut. Um diese Versorgung während der COVID-19-Pandemie weiterhin sicherzustellen, waren die Einführung gewisser Schutzmaßnahmen sowie intensivierte Telekommunikation innerhalb der SAPV erforderlich. Die vorliegende Befragung beschäftigt sich mit den Erfahrungen der betroffenen Palliativpatienten bzgl. der Pandemie und den damit einhergehenden Veränderungen in der Versorgung mittels SAPV. Besonderes Augenmerkt wurde hierbei auf Hygiene- und Schutzmaßnahmen sowie Telemedizin gelegt.

## Einleitung

Anfang 2020 wurde der erste Fall einer COVID-19-Infektion in Deutschland beschrieben [12]. Zu den Risikogruppen für schwere Verläufe gehören Menschen mit Komorbiditäten und einem geschwächten Immunsystem [20], somit insbesondere auch Palliativpatienten. Für die Versorgung von Palliativpatienten im ambulanten Bereich stehen in Deutschland die allgemeine ambulante Palliativversorgung (AAPV) und die spezialisierte ambulante Palliativversorgung (SAPV) zur Verfügung, wobei die von der SAPV versorgten Patienten meist von ihren Hausärzten weiter mitbetreut werden. Zu Beginn der Pandemie konnte die hausärztliche Versorgung, einschließlich der Haus- und Pflegeheimbesuche, nur noch deutlich reduziert erfolgen, da die Hausärzte entweder selbst infiziert oder überlastet waren [5, 25]. Um weiterhin eine adäquate Versorgung durch die SAPV zu gewährleisten, stand der Schutz des Personals vor einer COVID-19-Infektion im Vordergrund. Die geforderten Schutzmaßnahmen (z. B. Masken/Schutzkleidung, reduzierte Besprechungszeiten, ausreichende Distanz zu den Patienten, Reduktion der Hausbesuche und der Verweildauer des SAPV-Personals im häuslichen Umfeld [9]) bedeuteten eine Verringerung des persönlichen Kontakts mit einer entsprechenden Abnahme der Kommunikationsmöglichkeiten. Insbesondere Patienten in Pflegeheimen, die von den erforderlichen Sicherheitsvorkehrungen besonders betroffen waren, konnten nur noch eingeschränkt betreut werden. Um die persönliche Kommunikation mit den Patienten aufrechtzuerhalten, kam in der teilnehmenden SAPV die Telekommunikation verstärkt zur Anwendung. Während zum Zeitpunkt der Untersuchung mehrere Fachgesellschaften Empfehlungen zur Versorgung von Palliativpatienten in der Pandemie veröffentlicht haben, existierten nur wenige Daten über die persönlichen Erfahrungen von SAPV-Patienten während der Pandemie. In der vorliegenden Studie sollen die Erfahrungen und Probleme von SAPV-Patienten während der Pandemie anhand von Telefoninterviews untersucht werden. Dabei werden insbesondere die generelle Wahrnehmung der Patienten hinsichtlich der erforderlichen Hygiene- und Schutzmaßnahmen sowie deren Erfahrungen mit und ihre Vorstellungen von telemedizinischen Kommunikationsmöglichkeiten im Rahmen der SAPV-Versorgung berücksichtigt.

## Material und Methoden

Ende Februar 2020 führte wegen steigender COVID-19-Infektionszahlen die an der Studie beteiligte SAPV neue verbindliche Hygiene- und Schutzmaßnahmen ein. Dazu gehörten: Tragen von Schutzkleidung und -masken (FFP2), keine gemeinsamen Besuche von Arzt und Pflegepersonal, Checkliste mit definierten Fragen bzgl. einer COVID-19-Infektion vor Betreten der Patientenwohnung, weitestgehende Reduzierung von Verwandtenkontakten, Verringerung von Hausbesuchen sowie Nutzung moderner Kommunikationstechniken. Die SAPV-Koordinatoren prüften 168 Patienten (83 m, 85 w), die im Zeitraum vom 20.3. bis zum 20.04.2020 von der SAPV betreut wurden, auf die im Folgenden aufgeführten Auswahlkriterien und luden die passenden Patienten telefonisch zur Teilnahme an einem Telefoninterview ein.

Auswahlkriterien:

1. Palliatives Stadium der Erkrankung
2. Alter ≥ 18 Jahre
3. SAPV-Betreuung: 20.3. bis 20.04.2020
4. Regelmäßige Hausbesuche oder Videokonsultationen durch SAPV
5. Ausreichende physische und psychische Ressourcen zur Teilnahme am Interview

Ein aus Palliativmedizinern und Palliativ-care-Schwestern bestehendes Gremium entwickelte basierend auf den klinischen Erfahrungen unter Berücksichtigung der „GESIS survey guidelines“ [17] einen halbstrukturierten Interviewleitfaden, der aus 19 offenen Fragen bestand (Tab. 1). Die teilnehmenden Patienten wurden umfassend über die Studie informiert. Vor dem Interview wurde eine schriftliche Einwilligung der Teilnehmer eingeholt. Die Dokumentation und Auswertung erfolgten anonym. Die durchschnittliche Interviewdauer betrug 30 min ohne zeitliche Begrenzung, um ausreichend Zeit zur Beantwortung der Fragen zu geben. Das Institut für Marktforschung im Gesundheitswesen München führte die Interviews im Zeitraum von Juni bis August 2020 durch.

**Table 1:** 

Frage 1	Woran denken Sie zuerst, wenn Sie die Worte „COVID-19-Pandemie“ hören?
Frage 2	Wie kommen Sie persönlich mit der COVID-19-Pandemie zurecht?
Frage 3	Was war oder ist für Sie das größte Problem während der COVID-19-Pandemie?
Frage 4	Hatten oder haben Sie Angst davor, aufgrund Ihrer (Krebs‑)Erkrankung im Krankenhaus bleiben zu müssen?
Frage 5	Hatten Sie Angst oder Bedenken, während der COVID-19-Pandemie allein gelassen zu werden?
Frage 6	Wie hat sich Ihre SAPV durch die Pandemie verändert?
Frage 7	Was ist jetzt anders als vor der Pandemie?
Frage 8	Gab es während der COVID-19-Pandemie genügend Zeit für Gespräche mit den SAPV-Mitarbeitern?
Frage 9	Fühlten oder fühlen Sie sich bei der SAPV angemessen und sicher betreut?
Frage 10	Haben die Sicherheitsvorkehrungen (Hygiene etc.), die durch COVID-19 erforderlich wurden, Ihre Beziehung zu den SAPV-Mitarbeitern belastet oder haben Sie sich dadurch unsicher gefühlt?
Frage 11	Hatten oder haben Sie Angst, sich bei den SAPV-Besuchen mit COVID-19 zu infizieren?
Frage 12	Glauben Sie, dass die Patienten auch in einer Krise wie der COVID-19-Pandemie angemessen durch die SAPV versorgt werden können?
Frage 13	Hat sich Ihr Kontakt/Kommunikation mit der SAPV aufgrund der COVID-19-Pandemie verändert? Wenn ja, wie?
Frage 14	Was würden Sie gerne an Ihrer derzeitigen Kommunikation mit der SAPV ändern?
Frage 15	Haben Sie schon einmal an einer Videokonsultation mit SAPV-Mitarbeitern teilgenommen? Wenn ja: Wie sind Sie damit zurechtgekommen? Wenn nein: Ist Ihnen bekannt, dass es diese Möglichkeit gibt?
Frage 16	Was halten Sie persönlich von diesen modernen Kommunikationsformen?
Frage 17	Kann Ihrer Meinung nach eine Videoberatung gelegentlich einen Hausbesuch ersetzen?
Frage 18	Was ist für Sie persönlich in Zeiten der COVID-19-Pandemie besonders wichtig, wenn Sie von SAPV besucht werden?
Frage 19	Welche Folgen/Erfahrungen im Zusammenhang mit der COVID-19-Pandemie sollten bei der künftigen SAPV stärker berücksichtigt werden?

*COVID* Corona Virus Disease, *SAPV* Spezialisierte ambulante Palliativversorgung

Semi-strukturierte qualitative Interviews basieren auf offenen Gesprächen. Dies gewährleistet eine Atmosphäre, in der die Befragten alle relevanten Meinungen und Gedanken zum Thema äußern können, was zu aussagekräftigen, für die jeweilige Zielgruppe typischen Ergebnissen führt [8, 11, 16, 18].

Die Interviews wurden aufgezeichnet und für die weitere Bearbeitung transkribiert. Die Auswertung der Antworten erfolgte mit der Methode der qualitativen Inhaltsanalyse nach Mayring [18]. Dabei handelt es sich um ein mehrstufiges, strukturiertes, reproduzierbares Analyseverfahren, dessen Schritte die Kategorisierung, Kodierung, Neubewertung und Analyse der Interviews umfassen und so die qualitativen Daten systematisch und transparent abstrahieren. Nach einer ersten Sichtung des Materials wurden die Interviewfragen als Hauptkategorien festgelegt. Anschließend wurden für jede Hauptkategorie Unterkategorien gebildet, indem die wichtigsten Themen zusammengefasst und identifiziert wurden. Alle Interviewdaten wurden dann anhand des Kategoriensystems sortiert und tabellarisch dokumentiert (Kodierung). Dabei wurden die Kategorien immer wieder neu bewertet und angepasst, wenn sich neue Erkenntnisse ergaben. Schließlich wurden die Ergebnisse zusammengefasst und ausgewertet. Darüber hinaus wurden wörtliche Aussagen (Zitate) der Befragten in anonymisierter Form in die Analyse einbezogen, die die Denk- und Ausdrucksweise der Befragten verdeutlichen.

Ziel der qualitativen Interviews war es, folgende Punkte aus Sicht der Palliativpatienten näher zu untersuchen:

- Größtes Problem während der Pandemie
- Qualität der SAPV
- Angst vor Infektion durch die SAPV
- Angst, alleine gelassen zu werden
- Angst vor möglichem Krankenhausaufenthalt
- Teilnahme und Bewertung von Videokonsultationen mit der SAPV
- Folgen von Sicherheitsvorkehrungen in Bezug auf das Verhältnis zur SAPV
- Gesprächsmöglichkeiten mit SAPV-Mitarbeitern während der Pandemie

## Ergebnisse

Von den 36 ausgewählten Patienten nahmen 20 an den Interviews teil, 9 lehnten ab, 1 Patient konnte wegen Sprachproblemen nicht teilnehmen. Im Verlauf der Studie mussten 6 Teilnehmer ihre Einwilligung wegen akuter Verschlechterung ihres Gesundheitszustands zurückziehen. Die 20 teilnehmenden Patienten gliederten sich in folgenden Krankheitsgruppen auf: onkologisch/hämatologisch 18, kardial und neurologisch je 1. Das Durchschnittsalter betrug 77,5 Jahre.

Tab. 2 fasst die demografischen Daten der Patienten zusammen. 5 Palliativpatienten (2 m, 3 w; Durchschnittsalter 61,8 Jahre) nahmen (≥ 2 ×) an den Videosprechstunden teil.

**Table 2:** 

Wohnsitz	Geschlecht	Alter	Videokonsultation	Initialdiagnose/bisherige Therapie/Grund der Einweisung in die SAPV
Zuhause	W	67	Ja	ID: 7/11 Multiples Myelom/multiple antiproliferative Therapien/Fatigue, Schmerz
Hospiz	W	57	Ja	ID: 2/18 Glioblastom/Chirurgie und antiproliferative Therapie/Schmerz, Unruhe, Übelkeit
Pflegeheim	W	53	Ja	ID: 12/18 Ovarialkarzinom/Operation und antiproliferative Therapie/Infektionen, Schmerzen, Aszites
Zuhause	W	73	Nein	ID: 2/19 CUP/antiproliferative Therapie/Atemnot, Übelkeit, Angstzustände
Zuhause	W	49	Nein	ID: 5/16 Mammakarzinom/Operation, antiproliferative Therapie und Bestrahlung/Kurzatmigkeit, Schmerzen, Pleuraergüsse
Zuhause	M	63	Nein	ID: 11/18 Bronchialkarzinom/antiproliferative Therapie/Übelkeit, Schmerzen, Kurzatmigkeit
Zuhause	W	80	Nein	ID: 7/18 CUP/antiproliferative Therapie/Kurzatmigkeit, Pleuraergüsse
Zuhause	M	80	Nein	ID: 7/17 Bronchialkarzinom/antiproliferative Therapie/Übelkeit, Schmerzen, Kurzatmigkeit
Zuhause	M	88	Nein	ID: 11/08 Rektumkarzinom/Operation, Bestrahlung und antiproliferative Therapie/Kurzatmigkeit, Pleuraergüsse, Schmerzen
Zuhause	W	58	Nein	ID: 2/20 Glioblastom/Operation, Bestrahlung und antiproliferative Therapie/Schmerzen, Übelkeit, Verwirrung, Neigung zu Krämpfen
Zuhause	M	48	Nein	ID: 12/19 Neuroektodermaler Tumor/Operation/Müdigkeit, Schmerzen
Zuhause	W	77	Nein	ID: 10/17 Cholangiokarzinom/Operation und antiproliferative Therapie/Übelkeit, Pruritus
Zuhause	W	76	Nein	ID: 6/06 Bronchialkarzinom/antiproliferative Therapie/Kurzatmigkeit, Fieber, Schmerzen
Zuhause	M	90	Nein	ID: 12/18 MDS mit Übergang zu akuter Leukose/antiproliferative Therapie/Schmerzen, Blutungen, Angstzustände
Zuhause	M	77	Nein	ID: 12/16 Mantelzelllymphom/antiproliferative Therapie/Pleuraerguss, Aszites, Schmerzen, Übelkeit, Kurzatmigkeit
Zuhause	W	76	Ja	ID: 7/18 Endometriumkarzinom/Operation und antiproliferative Therapie/Pleuraerguss, Aszites, Schmerzen, Atemnot
Zuhause	M	44	Nein	ID: 8/17 ALS/medikamentöse Therapie/Atemnot, Spastik, Dysphagie, Angstzustände
Zuhause	M	90	Nein	ID: 7/12 Dekomp. Herzinsuffizienz/medikamentöse Therapie/Pleuraergüsse, Dyspnoe, Angstzustände
Zuhause	M	79	Nein	ID: 9/12 B‑Zell-Lymphom/antiproliferative Therapie/Schmerzen, Angstzustände
Zuhause	M	88	Ja	ID: 9/12 Prostatakarzinom/Operation, Hormontherapie und antiproliferative Therapie/Pleuraergüsse, Atemnot, Schmerzen

*ID* Initialdiagnose, *CUP* Cancer of unknown primary, *MDS* Myelodysplastisches Syndrom, *ALS* Amyotrophe Lateralsklerose

### Wahrgenommene Ängste und Befürchtungen

Die 20 befragten Patienten verbanden mit den Worten „COVID-19-Pandemie“ vor allem persönliche Einschränkungen und emotionsgeladene Gedanken wie Angst/Panik, z. B. vor Infektion oder weltweiter Ausbreitung. Sie verbanden damit Gedanken an Sterben und Einsamkeit. 17 Patienten gaben an, dass sie trotz aller Einschränkungen zurechtkamen: Sie blieben zu Hause, hielten sich an die Regeln und wurden von Familienmitgliedern unterstützt:

Ich bin die ganze Woche zu Hause, ich gehe nicht aus. Mein Sohn macht meine Einkäufe.

Das Hauptproblem der Pandemie war für die Befragten die Einsamkeit aufgrund des reduzierten Kontakts mit Familien/Freunden:

Dass ich mich nicht mit meinen Freunden treffen darf, dass wir uns nicht umarmen dürfen, das ist für mich mit Abstand das Schlimmste.

Die Patienten hatten Probleme mit den Einschränkungen im täglichen Leben, den strengen Hygienemaßnahmen und dem ständigen Tragen von Masken (z. B. Atemnot).

Die Gesichtsmaske und das ständige Desinfizieren.

Das Infektionsrisiko und die Gefahr einer zweiten Infektionswelle lösten bei einigen Befragten erhebliche Bedenken aus:

Dass es so weitergeht, weil sich viele Leute nicht an die Regeln halten, da mache ich mir Sorgen, dass es eine zweite Welle geben wird.

13 Patienten waren in Bezug auf COVID-19 besorgt bzgl. eines erforderlichen Krankenhausaufenthaltes, während 7 Befragte der Meinung waren, dass die Schutz- und Hygienemaßnahmen dort angemessen befolgt würden. 18 Befragte hatten keine Angst, während der Pandemie allein gelassen zu werden. Sie fühlten sich von Familienmitgliedern und SAPV-Mitarbeitern ausreichend unterstützt. 2 Patienten befürchteten aufgrund der steigenden Krankheitsfälle einen Mangel an medizinischer Versorgung. Nur 3 der 20 Patienten gaben keine pandemiebedingten Probleme an.

### Änderungen in der Betreuung durch die SAPV

Die Befragten gaben pandemiebedingte Änderungen in der SAPV durch das Tragen von Schutzkleidung und Gesichtsmasken an.

SAPV kommt mit einer Maske und mit Schutzkleidung.

Einige Patienten gaben eine Zunahme der telefonischen Kontakte an, während gleichzeitig die persönlichen Kontakte reduziert wurden.

Die SAPV kam seltener persönlich, die Dinge wurden telefonisch erledigt.

2 Befragte nannten als Veränderung die Einführung von Videosprechstunden, 4 gaben keine Veränderung an. Alle Patienten bestätigten ausreichende Kommunikationsmöglichkeiten mit der SAPV und fühlten sich sicher und angemessen betreut.

### Zufriedenheit mit der angepassten SAPV-Struktur

20 befragte Palliativpatienten gaben an, dass die aufgrund von COVID-19 getroffenen Sicherheits- und Hygienemaßnahmen (Masken, Schutzkittel, Handschuhe, Distanzierungsregeln, Desinfektion) das Verhältnis zwischen Patienten und SAPV-Personal nicht belasten oder verunsichern. Sie halten diese Sicherheitsmaßnahmen für sehr wichtig. Keiner der 20 Befragten hatte Angst vor Ansteckung durch das SAPV-Personal, da die Schutzmaßnahmen der SAPV ihnen ein starkes Sicherheitsgefühl vermittelte.

Nein, sie kamen immer mit Kittel und Maske ausgestattet und hielten Abstand.

Dabei war das durchgehende Tragen von Masken/Schutzkleidung am wichtigsten und die Einhaltung der Hygienemaßnahmen am zweitwichtigsten.

Die Schutzmaßnahmen müssen eingehalten werden.

Für 4 Patienten stand der persönliche Kontakt im Vordergrund.

Der persönliche Kontakt ist wichtig und muss aufrechterhalten werden.

Alle 20 Patienten waren der Meinung, dass sie durch die SAPV auch in einer Krise wie COVID-19 angemessen und sicher versorgt werden können.

12 Befragte wünschten keine Fortführung pandemiebedingter Strukturänderungen der SAPV nach Beendigung derselben, 5 wünschten die ständige Einhaltung von Schutzmaßnahmen.

### Moderne Telekommunikationsmöglichkeiten aus Patientensicht

Bei 11 von 20 Befragten änderte sich der Kontakt/die Kommunikation mit der SAPV während der Pandemie nicht. 2 Patienten erhielten weniger Besuche und stattdessen mehr Telefonanrufe/Videosprechstunden. 5 Patienten waren mit den Kommunikationskanälen zufrieden und konnten keine Änderungen feststellen, da sie während der Pandemie in die SAPV aufgenommen wurden und damit Vergleichsmöglichkeiten fehlten. 17 Patienten wünschten keine Änderungen an der derzeitigen Kommunikation mit der SAPV. Ein Patient zog persönliche Besuche dem telefonischen Kontakt vor. Während des Zeitraums dieser Studie nahmen 15 Befragte noch nicht an der Videosprechstunde teil. Von diesen 15 wussten 11, dass es diese Möglichkeit gibt. Die fünf Patienten, die Erfahrung mit Videosprechstunden hatten, kamen mit Unterstützung der Pflegeperson oder des Ehepartners gut mit diesem Medium zurecht.

Zehn Befragte standen der Videosprechstunde positiv gegenüber und hielten diese Option für nützlich und interessant:

Ich muss sagen, dass ich das auf jeden Fall für eine gute Möglichkeit halte; in Schweden gibt es das schon seit einiger Zeit. Ich persönlich hasse Videokonferenzen, ich bin nicht wirklich der Typ dafür. Aber wenn die Möglichkeit besteht und es Sinn macht, würde ich es auf jeden Fall machen.

4 Befragte standen der Videosprechstunde kritisch gegenüber und lehnten diese Form des Kontakts ab. 4 Patienten waren zwiespältig, und zwei Befragte konnten sich wegen mangelnder Erfahrung nicht dazu äußern. 13 Befragte waren der Meinung, dass eine Videosprechstunde den Hausbesuch des Arztes nicht ersetzen kann – nicht einmal gelegentlich. 4 Befragte zogen diese Art der Kommunikation durchaus als Alternative in Betracht. 3 Befragte hielten diese Option für denkbar, machten ihren Einsatz aber von Voraussetzungen, wie dem Inhalt des Gesprächs, abhängig.

## Diskussion

COVID-19 hat zu einschneidenden Veränderungen in der ambulanten Palliativversorgung geführt und eine zusätzliche Belastung für schwerstkranke Patienten mit sich gebracht. Die vorliegende interviewbasierte Studie beleuchtet die persönlichen Probleme und Erfahrungen von Palliativpatienten mit einer an COVID-19 angepassten SAPV zu Beginn der Pandemie. Die Verteilung der Diagnosen in der untersuchten Patientenkohorte entsprach der üblichen Verteilung in einem SAPV-Setting (Tab. 2; [13]).

Da soziale Isolation nicht nur die Lebensqualität [21], sondern auch das Fortschreiten der Krankheit fördern kann [14], war die untersuchte Patientengruppe besonders von anhaltenden Kontaktverboten betroffen [2]. Einsamkeit war neben der Angst vor Infektion die Hauptsorge der befragten Patienten. Neben dem Kontakt zu Angehörigen war der persönliche Kontakt zur SAPV für die Patienten besonders wichtig.

Um die Versorgung von Palliativpatienten mit der SAPV aufrechtzuerhalten, wurden im Laufe der Pandemie sowohl international [4] als auch in Deutschland – hier durch die Bundesarbeitsgemeinschaft Spezialisierte Ambulante Palliativversorgung (BAG-SAPV) in Zusammenarbeit mit der Deutschen Gesellschaft für Palliativmedizin (DGP) sowie dem Forschungsverbund Palliativmedizin im Netzwerk Universitätsmedizin – Handlungsempfehlungen für SAPV-Teams während der Pandemie veröffentlicht [1, 9]. Sie beinhalten u. a. – wie von der teilnehmenden SAPV durchgeführt – eine reduzierte Anzahl und Dauer von Hausbesuchen. Dennoch fühlten sich die meisten Patienten weiterhin von der SAPV angemessen und sicher betreut. Die zunehmende Nutzung der Telemedizin, wie sie auch im aktuellen Projekt „PALLPAN“ beschrieben wird [1], mag dazu beigetragen haben. Gemäß den Empfehlungen der S1-Leitlinie der Deutschen Gesellschaft für Schmerztherapie (DGS), die insbesondere den Eigenschutz gegenüber dem Fremdschutz betont [22], hat die SAPV seit Beginn der Pandemie entsprechend adäquaten Schutzmaßnahmen, d. h. FFP2-Masken, Kittel, Handschuhen, Einhaltung der Distanzierungsregeln und Hygienemaßnahmen, höchste Priorität eingeräumt. Aufgrund mangelnder Ressourcen, insbesondere zu Beginn der COVID-19-Pandemie, musste jedoch oft auf behelfsmäßige Schutzausrüstung zurückgegriffen werden [15].

Ähnlich wie viele Krebspatienten [6] äußerten einige SAPV-Patienten Angst vor einem Krankenhausaufenthalt aufgrund der Möglichkeit einer COVID-19-Infektion, waren aber nicht besorgt, vom SAPV-Team angesteckt zu werden. Obwohl die notwendigen Distanzierungsregeln und die Schutzkleidung den persönlichen Kontakt und die klinische Beurteilung des Patienten während der Hausbesuche der SAPV beeinträchtigten (u. a. fehlende Nähe und Mimik [24]), führten diese Maßnahmen zu einem starken Sicherheitsgefühl bei den Patienten, sodass der Nachteil des eingeschränkten persönlichen Kontakts durchweg akzeptiert wurde. Während der Einsatz von Videokonferenzen und Telekommunikation im Bereich der allgemeinen Patientenversorgung stark zunahm [19] und zum Versorgungsstandard wurde [2], betrachteten die Palliativpatienten diese neue Kommunikationstechnologie während der Pandemie eher als Notlösung und zogen den persönlichen Kontakt mit Ärzten/Pflegepersonal vor. Die Telekommunikation trug dazu bei, dass die Patienten mit der SAPV durchweg zufrieden waren, obwohl die Hausbesuche aufgrund von Schutzmaßnahmen und einer steigenden Zahl von Patienten in der teilnehmenden SAPV eingeschränkt waren.

Telemedizin bietet eine zusätzliche Möglichkeit, Palliativpatienten auch in Krisensituationen adäquat zu versorgen [2, 3]. Inwieweit die Telemedizin jedoch einen nachweisbaren Nutzen in der Versorgung von Palliativpatienten hat, bleibt aufgrund der wenigen Studienergebnisse in diesem Versorgungsbereich offen [1, 10]; weitere Studien werden in Zukunft durchgeführt werden müssen [23].

## Limitationen

Es wurden Patienten nur einer SAPV befragt, weshalb keine Verallgemeinerung der Ergebnisse möglich ist. Bei den Befragten handelte es sich um Patienten, die sich bereits in einer weit fortgeschrittenen palliativen Situation befanden. Diese von der SAPV versorgte Patientengruppe entspricht nur circa 10 % aller Palliativpatienten [7], sodass die vorliegenden Patientenaussagen nur bedingt auf die Gesamtzahl der Palliativpatienten bezogen werden können. Alle befragten Palliativpatienten stammten aus ländlichen Gebieten, in denen insbesondere die Durchführung von Videosprechstunden aufgrund fehlender technischer Infrastruktur oft erschwert war. Die Ergebnisse von telemedizinischen Befragungen können insbesondere dann abweichen, wenn sie z. B. in Großstädten durchgeführt werden. Nur fünf der befragten Patienten hatten zum Zeitpunkt der Befragung an einer Videosprechstunde teilgenommen, sodass dieser Teil der Telekommunikation nur bedingt ausgewertet werden konnte.

## Fazit für die Praxis

- Auch während der Pandemie können Palliativpatienten durch die SAPV zufriedenstellend und sicher versorgt werden.
- Die Sicherheitsvorkehrungen sollten konsequent eingehalten werden, da sie dazu beitragen können, die Ängste der Patienten zu reduzieren.
- Der reduzierte Kontakt durch eine geringere Anzahl von Hausbesuchen kann zumindest teilweise durch Telekommunikation kompensiert werden. Der Kontakt sollte jedoch häufig aufgenommen werden, um der Isolation der Palliativpatienten entgegenzuwirken, die durch COVID-19 und die damit verbundenen Folgen zugenommen hat.
